# Initial Evaluation of Acceptability, Engagement, and Effectiveness of the MO App to Provide Tailored and Comprehensive Support for Smoking Cessation: Development and Usability Study

**DOI:** 10.2196/55239

**Published:** 2024-10-29

**Authors:** Shuo Zhou, Paul Brunetta, Joshva Silvasstar, Greg Feldman, Nicolas Oromi, Sheana Bull

**Affiliations:** 1 Department of Communication Studies School of Communication and System Health Lab Hong Kong Baptist University Hong Kong China (Hong Kong); 2 Fontana Tobacco Treatment Center University of California San Francisco San Francisco, CA United States; 3 Department of Community and Behavioral Health, mHealth Impact Lab Colorado School of Public Health University of Colorado Aurora, CO United States; 4 Mobile Applications for Connected Health Lakewood, CO United States; 5 Ingenious Agency Montevideo Uruguay

**Keywords:** smoking cessation, tobacco, mobile phone app, mHealth, mobile health, iterative design, feasibility, acceptability, engagement, efficacy, mobile phone

## Abstract

**Background:**

Despite the growing availability of smoking cessation apps, low engagement and cessation rates have remained a significant challenge. To address this issue, we used a user-centered design to iteratively develop a mobile app (MO) to provide comprehensive, tailored, and evidence-based content to support smokers in their quitting journey.

**Objective:**

This study examined the acceptability, use, and preliminary efficacy of the MO app for smoking cessation. Specifically, we sought to understand smokers’ preferred features, engagement, and satisfaction with MO; identify concerns in using the app and ways to improve the app; and evaluate its smoking cessation outcomes.

**Methods:**

Through 3 cohorts, we recruited 10, 12, and 85 adult smokers who attempted to quit smoking to pilot-test the MO app between December 2019 and July 2022. Participants were instructed to complete a baseline survey, interact with the app for 6 weeks, and fill in a postsurvey at week 6. Participants in cohort 3 completed an additional postsurvey at week 12. Participants’ app use was tracked and analyzed. The primary outcome measures were participants’ 7-day point prevalence abstinence at 6 and 12 weeks.

**Results:**

Participants reported high levels of satisfaction with the MO app across all 3 cohorts, rating it between 4.40 and 4.76 on a scale of 5 for acceptability. Users engaged with app activities for an average of 89 to 159 times over 35 days. The most liked features of the app included “quit plan,” “tracking,” “reminders and notifications,” “MOtalks,” and “motivational quotes.” The 7-day point prevalence abstinence rate of the modified intention to treat population in cohort 3 was 58% at 6 weeks and 52% at 12 weeks. Those who interacted more frequently with app features and engaged with more diverse activities were more likely to maintain abstinence at weeks 6 and 12. For each additional time logged into the app, the odds of staying abstinent at week 12 increased by 5% (odds ratio [OR] 1.05, 95% CI 1.01-1.08). Participants who earned >5000 points during app use also had higher odds of quitting at both 6 weeks (OR 3.12, 95% CI 1.25-7.75) and 12 weeks (OR 4.65, 95% CI 1.83-11.76), compared with those who earned <5000 points.

**Conclusions:**

Our study demonstrated that MO is a feasible mobile phone app with high acceptability and usability and can effectively deliver smoking cessation support to individuals who want to quit. Implications for developing and evaluating mobile phone apps for smoking cessation are discussed.

## Introduction

### Background

Tobacco use remains the leading cause of preventable death in the United States and globally, with nearly half a million people dying each year in the United States alone due to tobacco-related diseases. Over a billion people worldwide still smoke [1], which highlights the enormous global burden of tobacco use. Recent evidence indicates that quitting before the age of 40 years can reduce the risk of premature death by 90%. This underscores the importance of effective tobacco cessation interventions. Cessation at any age can provide major disease risk reduction benefits. Despite the increasing awareness of the adverse health effects of smoking and the clear benefits of quitting, cessation rates remain low, particularly among highly nicotine-dependent individuals who face a high risk of relapse [2]. Nicotine is one of the most addictive substances known in medicine, which explains the major challenges of successful quitting and contributes to the persistently low cessation rates. Thus, there is a critical need for accessible and clinically validated smoking cessation interventions that can effectively address the barriers and challenges associated with nicotine addiction.

Compared with spontaneous unmedicated cessation rates (about 2%/year), nicotine cessation therapies have documented success rates of approximately 20% at 1 year. Motivated smokers who receive professional counseling and evidence–based cessation medications can have a success rate as high as 35% at 1 year [2,3]. However, traditional counseling interventions have limited reach, and the multiple available medications for cessation can be confusing for people seeking help. Patients face various barriers to accessing face-to-face tobacco cessation services, including financial challenges, language issues, traveling difficulties, and low-time commitment for in-person counseling [4,5]. The social stigma for free telephone-based counseling [6], lower-income patients’ distrust of health care systems [7], and low awareness of the health risks of smoking further exacerbate these challenges for smokers with low socioeconomic status (SES). Hence, there is an urgent need to disseminate high-quality cessation treatments to the widest possible target audiences, including those in remote locations, those without health insurance, and those who cannot or will not receive counseling in person. Innovative strategies that deliver evidence–based cessation interventions to smokers attempting to quit could further enhance the success rate of quitting and prevent tobacco-related diseases while improving both physical and psychological well-being. Therefore, exploring novel approaches to promote smoking cessation is a critical public health priority.

### Mobile Apps for Smoking Cessation

The ubiquity and widespread availability of smartphones provide an opportunity to extend the reach of smoking cessation professional counseling and evidence-based treatment beyond clinics. As of 2021, 85% of the adults in the United States own a smartphone, which covers low-income populations who are known to have higher smoking rates. In 2017, mobile health (mHealth) apps were downloaded 3.7 billion times worldwide [8], and this number increased dramatically during and after the COVID-19 pandemic. The high demand for mHealth apps represents a promising opportunity to leverage mobile technologies to facilitate smoking cessation and reduce health disparities in quitting.

Increasing evidence has shown the positive outcomes of mobile phone apps for smoking cessation [9-15]. According to a review, the cessation rate of people using mobile apps ranges from 13% to 24%, higher than the average cessation rate of using SMS text messaging alone (10%) [16]. Combining smartphone-based interventions with pharmacotherapy yields greater smoking abstinence rates than using pharmacotherapy alone [17]. However, limited and mixed evidence exists for the long-term effectiveness and retention rates of mobile phone apps. Very few of the popular smoking cessation apps (4% of the top 50 apps) are evidence based and adhere to the US Public Health Service’s Clinical Practice Guidelines for Treating Tobacco Use and Dependence [18-21]. Currently, available apps lack tailored features and integration of multiple evidence–based approaches into the app design, such as a comprehensive review of medication options. In addition, existing apps have limited support throughout the different phases of smoking cessation, including preparation to quit, maintenance of cessation, and relapse prevention and support.

We developed a comprehensive and integrative smoking cessation mobile phone app called “MO”—an abbreviation for “mobile” and “motivation”—to address these limitations. The aim of MO is to provide user-centered, tailored cessation support using evidence-based approaches, integrating cognitive-behavioral therapy, peer support, and medication information. MO provides learning materials regarding how to quit smoking in different formats (eg, texts, audios, videos, and quizzes) and explains in easy-to-understand language the intensely addictive nature of nicotine. The app offers cessation and relapse support throughout the “prequit and planning,” “quit and maintenance,” and “relapse prevention” phases of smoking cessation and helps the user remain motivated toward quitting. Existing smoking cessation apps often have low user engagement and retention rates. MO seeks to address this by consistently motivating users to quit and encouraging long-term engagement to increase the efficacy of quitting.

### Development of MO Smoking Cessation App

The MO app was developed with guidance from the US Public Health Service’s Clinical Practice Guidelines for Treating Tobacco Use and Dependence, using the 5 *A*’s model (ask, advise, assess, assist, and arrange) and more than 80 evidence–based behavioral modification and motivation techniques. The app was designed to guide users step-by-step throughout their quitting process.

In the prequit or planning phase, there are 3 main functions: creating an individualized profile, tracking smoking behaviors, and advising on tasks and activities to prepare for quitting. The app’s first function (setting up profile) assesses factors, such as level of nicotine addiction, health conditions, use of medications, quit date, motivations, and reasons for quitting, gathered from user profile information to make tailored recommendations based on proprietary algorithms and using tenets of motivational interviewing. The second function (tracking behaviors) is to help users track smoking behaviors (eg, concurrent activities, locations of smoking, time of the day, and the number of cigarettes smoked), identify triggers, and guide them through the process of choosing substitutive activities or distractions to replace smoking. The third function (task advising and learning) is to prepare users to stop smoking through education and specific activities, such as obtaining medications, modifying their environment, and informing family and friends to support their quit effort. General advice and strategies on how to stop smoking are available in text, audio, and video formats for users to learn at their own pace. Each day before the quit day, users can use this function to go through a specific theme preparing them to quit smoking. Cessation medications are described in detail and consistent with existing guidelines, yet none are specifically recommended, and the user is encouraged to make the decision about medication use with a health care provider.

In the quit or maintenance phase, the app also has 3 main functions. The first is the daily check-in, which prompts the users to track their moods, urges, smoking behavior, use of medications, and interactions with their social network. The second function is the “urge” button, which provides immediate support and strategies for managing urges to smoke. The third function delivers educational content through video clips and quizzes based on user motivations and data gathered in the prequit phase. The smoker will guide themselves through content that matches their highest interest and view new modules or review old modules to reinforce knowledge about smoking cessation.

The app also includes functions to manage lapses (a momentary slip), relapses (return to smoking that may last for several days before resuming the cessation program), and collapses (a major reduction in motivation and return to smoking), which are often neglected by smoking cessation apps. The MO app anticipates these events, supports users through these inevitably difficult moments, and keeps users engaged and motivated. Specific functions for this phase include educational videos and audio segments helping users determine what happened, reestablish motivations, and how to prevent it the next time.

The app incorporates several features to encourage long-term user engagement. Every time a user opens the app, an uplifting quote will pop up to boost smokers’ motivation to quit. Gamification features, including a point system and badges, help users celebrate their success in the quitting process. This feature visualizes users’ progress and motivates them to stay abstinent. As each user completes a module, they will receive certain points (250, 500, or 1000) for the activity they engaged in. These points are designed to recognize the actions users take toward the quitting goal. Modules that are reviewed can be repeated to accumulate additional points. This “nudge” feature assigns more points to activities that have scientific evidence suggesting a positive role in cessation. Points are shown on the home screen, which may help users feel invested in their progress and remind them of their previous efforts if a lapse or relapse occurs after the quit date. Existing evidence has consistently suggested that financial incentives can improve smoking cessation outcomes [22]. As a potential reward, users who obtained >10,000 points will enter a lottery for a US $50 gift card in addition to the study compensation.

Informed by text-to-quit data suggesting social group aggregation may improve outcomes [23], the MO app has an internal chat feature that allows daily posting to the quitter’s support network. This is a secure, customizable platform uniquely created for the app. The chat function allows for secure data management and professional moderation, with the goal to provide a supportive, understanding community breaking the negative effects of isolation and motivating users through positive social contagion. In addition, the story booth feature allows users to record and share brief video clips about their cessation journey and to view other people’s stories.

**Figure 1:**
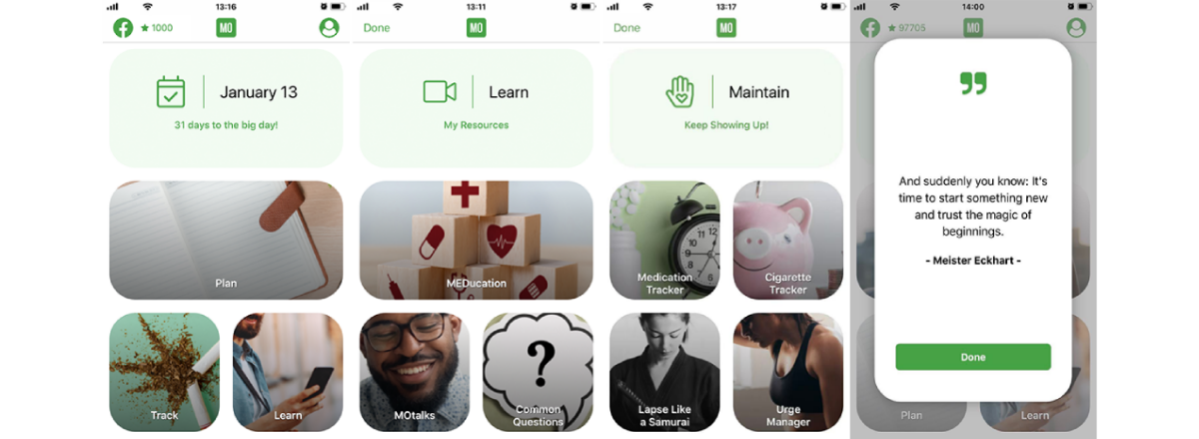
Interface and main features of the MO smoking cessation app.

The main features of the MO app are illustrated in Figure 1. The development of MO involved a web-based asynchronous focus group with end users to evaluate and provide suggestions for the initial prototypes of the app. Participants were asked to post comments to a web-based discussion board as to the viability, content usability, and attractiveness of each app element and the ability for each to engage the intended audience. We incorporated participants’ comments and developed the beta version of the MO smoking cessation app.

### Research Questions and Hypotheses

This study used an iterative process to evaluate and improve the feasibility and usability of the beta version of the app. A series of studies were conducted to address the following research questions (RQs) and hypotheses related to the app’s acceptability, user engagement, and preliminary effectiveness on smoking cessation.

- RQ1: Are participants satisfied with using MO for smoking cessation?
- RQ2: What are the most liked features of the app?
- RQ3: What are the least liked features of the app?
- RQ4: What are the suggestions for improving the app?
- RQ5: What are the 7-day point prevalence abstinence rates at 6 and 12 weeks after using the MO app?
- Hypothesis 1: Participants who engage with a greater number of app activities are more likely to self-report quitting at the 6 and 12-week follow-ups.
- Hypothesis 2: Participants who earned more points are more likely to self-report quitting at the 6 and 12-week follow-ups.

## Methods

### Overview of the Study Design

Studies were conducted in 3 participant cohorts to examine the RQs and hypotheses mentioned earlier. In cohorts 1 and 2, we examined acceptability and usability of the app among a group of end users to ensure the various features work as intended, are perceived acceptable by the intended users, and can be easily used. Features and interfaces of the app will be refined based on participants’ use experience and feedback. In cohort 3, we additionally assessed the preliminary effectiveness of the MO app on smoking cessation outcomes.

### Participant Recruitment

We intended to recruit 10, 10, and 80 participants into cohorts 1, 2, and 3, respectively, during the following 3 periods: December 19, 2019, to March 19, 2020; August 10 to December 10, 2020; and April 7 to July 26, 2022. Eligible participants included (1) persons aged between 18 and 59 years; (2) who were current smokers consuming at least 5 cigarettes daily for at least the past 12 months, as consistent with cessation trials [24]; (3) self-reported a relatively high level of commitment to quit smoking of at least 7 on a scale of 0 to 10; (4) who were interested in learning skills to quit smoking; (5) resided in the United States; (6) had at least daily access to their own iPhone with internet access; (7) were able to read English; and (8) were not using other smoking cessation interventions (including apps, medications, or other intervention studies). We excluded participants who were younger than 18 years or older than 59 years as they may have had different cessation motivations; who had health contraindications to nicotine patch use; who were actively taking medication for depression, anxiety, or quitting smoking; who used illicit drugs or marijuana; or who failed to provide contact information.

Participants were recruited using web-based advertising through Craigslist (Craig Newmark) posts, Facebook (Meta), and Google (Google LLC) advertisements, and a university listserv for research participant recruitment. People who saw the advertisements could also refer the study information to their smoking relatives or friends. Interested participants clicked the enrollment link in the advertisements, which directed them to a short screening survey. Once eligibility was established, participants were shown a short video containing the informed consent materials.

Eligible participants who consented to participate were enrolled in the study. They were asked to complete a baseline survey to document their smoking history, use of nicotine replacement, and other health behaviors. We documented factors known to correlate with smoking cessation, such as social support, attitudes and norms related to smoking, and intentions to quit.

Participants were instructed to download MO and interact with the app and its various features for 6 weeks. A 10-minute walkthrough video was sent to every participant to introduce the main features of the app. The walkthrough video is available in Multimedia Appendix 1. A postsurvey was developed to examine app acceptability and usability and was sent to participants at 6 weeks (day 42) and at 12 weeks (day 84, only for cohort 3). Participants that returned the 6-week and 12-week questionnaires were part of a completer’s analysis of efficacy. Participants not returning the questionnaire were imputed as nonresponders and having returned to smoking.

### Measures

#### Acceptability

Acceptability refers to the degree to which an intervention is deemed acceptable by its intended users. In cohorts 1 and 2, we measured users’ acceptability of MO using 3 items: “I enjoyed using the MO smoking cessation app,” “The MO smoking cessation app was useful to me,” and “The MO smoking cessation app was easy to navigate” on a 5-point Likert scale (1=strongly disagree and 5=strongly agree). In cohort 3, we additionally asked 6 questions adapted from previous studies on app-based smoking cessation interventions [25] to have a more comprehensive evaluation of users’ satisfaction with and perceived usefulness of the MO app for smoking cessation.

#### App Engagement

We tracked participants’ interaction with and navigation through the MO app through Firebase Performance Monitoring (Google LLC) for 12 weeks. Specifically, we recorded the type of activities users engaged in within the app, whether they completed each module or task, and the frequency of engagement. We also asked users to identify their most liked and disliked app features, explain why they liked or disliked certain features, and provide suggestions for improving the app through open-ended survey questions.

#### Effectiveness

In cohort 3, smoking cessation outcomes were additionally examined. Participants were asked to self-report their smoking status at 6 and 12 weeks through 2 postenrollment surveys, including the 7-day point prevalence abstinence rate as the primary outcome variable, number of cigarettes smoked, and frequency of smoking in the past month. Self-report 7-day point prevalence abstinence, which is one of the commonly used measures for evaluating users’ smoking cessation outcome [26], asks whether participants maintain smoke-free in the past 7 days.

### Analytic Approach

The intent-to-treat (ITT) group typically includes all participants enrolled in the study who were assigned to receive a treatment. The modified ITT (mITT) is a subset of the ITT sample that removed participants who did not receive or start the treatment. In our study, participants who completed the screening test and successfully enrolled were considered the ITT group, and those who downloaded and used the MO app were considered the mITT group. The mITT group is more appropriate for the app engagement and preliminary efficacy analyses because they are the true participants who became exposed to and received the smoking cessation treatment, removing participants who did not download, open, or interact with the MO app at all. Because acceptability is a subjective evaluation of participants’ satisfaction with the app, we were only able to analyze data input by participants who completed the postsurvey. Those who dropped out of the study were not included in the acceptability analysis.

To examine acceptability (RQ1), we calculated participants’ average level of satisfaction with the MO smoking cessation app. We anticipated that at least 80% of the users will be “satisfied” or “very satisfied” with the app. To address RQs on usability (RQs 2-4), we qualitatively analyzed participants’ responses to open-ended questions—to identify themes around (1) difficulties in using or navigating the app, (2) liked features, (3) disliked features, and (4) suggestions for improving the app. We also evaluated engagement by calculating multiple aspects of user experience with the app, including the number of times the app was opened and the frequency and types of features used.

To explore RQ 5, we assessed the percentage of participants remaining abstinent from cigarette smoking and use of other nicotine or tobacco products in the previous 7 days at 6 and 12 weeks after using the app. All participants who completed the baseline survey, downloaded the app, and answered the questionnaire were included in this completer analysis. Participants that were lost to follow-up at the 6th and 12th weeks were imputed to be smoking.

We also examined the dose response relationship between quit status and greater use of the app in general (hypothesis 1) and more points earned (hypothesis 2) using logistic regressions to test the proposed hypotheses. We conducted exploratory analyses to assess the relationship between use of specific features of the app and the participant’s quit status.

### Ethical Considerations

The study was reviewed and approved by the Colorado Multiple Institutional Review Board (protocol number 14-2225). Participants were asked to view the informed consent video. Contact information for research staff was included in the video in case participants had any questions during the consent process. The material covered in the video was made available in a printable document so the participant could download and print it if desired. Participation in this study was completely voluntary. Participants may decline to participate or withdraw from the study at any time without penalty. Participants in cohorts 1 and 2 received a US $20 gift card incentive for participation. For cohort 3, participants received a US $40 gift card incentive for participation, as 2 follow-up surveys (at 6 and 12 weeks) were collected. All information was kept confidential, and the data were deidentified. Study procedures were in accordance with institutional ethical standards for conducting human subjects research and with the Helsinki Declaration.

## Results

### Participant Recruitment and Demographics

We enrolled 15, 19, and 132 eligible participants (the ITT groups) into the 3 cohorts. Among them, 9 (60%), 12 (63%), and 85 (64.4%) participants (the mITT group) downloaded and used the MO app. All statistics were calculated based on this mITT sample. The retention rates at 6-week follow-up were 90%, 83%, and 66%, respectively. For cohort 3, the retention rate at 12 weeks was 55%. Participants’ basic demographic characteristics, smoking history, level of commitment to quit smoking, and sources of recruitment were summarized in Table 1.

**Table 1:** Demographics of participants across the 3 cohorts.

Characteristic		Cohort 1 (n=9), n (%)	Cohort 2 (n=12), n (%)	Cohort 3 (n=85), n (%)
**Sex**				
	Male	5 (56)	3 (25)	66 (78)
	Female	4 (44)	9 (75)	19 (22)
**Age (years)**				
	18-30	4 (44)	2 (17)	34 (40)
	31-40	4 (44)	5 (42)	42 (49)
	41-50	1 (11)	2 (17)	7 (8)
	51-59	0 (0)	3 (25)	2 (2)
**Ethnicity**				
	Hispanic	3 (33)	0 (0)	7 (8)
	Non-Hispanic	6 (67)	12 (100)	78 (92)
**Race**				
	Black or African American	2 (22)	5 (42)	54 (64)
	White	6 (67)	6 (50)	30 (35)
	Other	1 (11)	1 (8)	1 (1)
**Age started smoking (years)**				
	13-17	4 (44)	7 (58)	12 (14)
	18-21	2 (22)	4 (33)	39 (46)
	22-30	2 (22)	1 (8)	30 (36)
	31-40	1 (1)	0 (0)	2 (2)
	41-50	0 (0)	0 (0)	1 (1)
**Smoking history (years)**				
	1-5	3 (33)	4 (33)	35 (41)
	5-10	4 (44)	4 (33)	33 (39)
	10-20	2 (22)	4 (33)	13 (15)
	>20	0 (0)	0 (0)	4 (5)
**Attempted to quit smoking in the past year**				
	Yes	3 (33)	6 (50)	61 (72)
	No	1 (11)	6 (50)	22 (26)
	Not sure	5 (56)	0 (0)	2 (2)
**Previous use of nicotine replacement**				
	Yes	—^a^	7 (58)	20 (24)
	Have used gum^b^	—	4 (33)	16 (19)
	Have used skin patches^b^	—	6 (50)	9 (11)
	Have used lozenge^b^	—	2 (17)	9 (11)
	Have used inhaler^b^	—	1 (8)	13 (15)
	No	—	5 (42)	65 (77)
**Parental smoking in childhood**				
	Yes	—	8 (67)	56 (66)
	No	—	4 (33)	25 (39)
	Not sure	—	0 (0)	4 (5)
**Be often around people who smoke (eg, family member, friend, or colleague)**				
	Yes	—	9 (75)	78 (92)
	No	—	3 (25)	7 (8)
**Often in social situations where others are smoking**				
	Yes	—	8 (67)	80 (94)
	No	—	4 (33)	4 (5)
	Not sure	—	0 (0)	1 (1)
**Commitment to quit smoking (0-10 scale)**				
	7	2 (22)	3 (25)	10 (12)
	8	0 (0)	1 (8)	13 (15)
	9	2 (22)	4 (33)	17 (20)
	10	5 (56)	4 (33)	45 (53)
**Sources of knowing this study**				
	Facebook	8 (89)	5 (42)	48 (56)
	Google	0 (0)	5 (42)	24 (28)
	Craigslist	0 (0)	0 (0)	8 (9)
	Friends	1 (11)	0 (0)	4 (5)
	Listserv	0 (0)	2 (17)	1 (1)

^a^Not available.

^b^These questions were only asked among participants who indicated previous use of nicotine replacement.

### Acceptability

Overall, participants had high levels of satisfaction with using the MO app. At 6 weeks, participants from the 3 cohorts reported 4.67, 4.40, and 4.76 out of 5 for app satisfaction. Table 2 shows details regarding participants’ acceptability of the app.

**Table 2:** Acceptability of the MO app across the 3 cohorts.

Statements		Cohort 1: 6 weeks, mean (SD)	Cohort 2: 6 weeks, mean (SD)	Cohort 3: 6 weeks, mean (SD)	Cohort 3: 12 weeks, mean (SD)
**Satisfaction**					
	I enjoyed using the MO smoking cessation app	4.63 (0.70)	4.60 (0.66)	4.86 (0.35)	4.87 (0.34)
	The MO smoking cessation app was useful to me	4.75 (0.43)	4.30 (0.64)	4.71 (0.49)	4.62 (0.80)
	The MO smoking cessation app was easy to navigate	4.63 (0.48)	4.30 (0.64)	4.70 (0.66)	4.62 (0.71)
**Satisfaction with the app for cessation treatment**					
	I can rely on the app to provide guidance that will help me to quit smoking and stay quit.	—^a^	—	4.59 (0.53)	4.70 (0.55)
	I feel that the app provides smoking cessation treatment that is personalized to my specific needs.	—	—	4.46 (0.71)	4.57 (0.58)
	I believe that the app will help me to quit smoking and stay quit.	—	—	4.54 (0.66)	4.55 (0.72)
	The app knows how to help me to quit smoking.	—	—	4.61 (0.53)	4.70 (0.47)
	I believe I can depend on the app.	—	—	4.46 (0.60)	4.47 (0.88)
	I find the app to be annoying.	—	—	1.31 (0.57)	1.48 (0.91)

^a^Not available.

### App Engagement

On the basis of the tracked data, Table 3 summarizes participants’ general use of the app and engagement with specific features. Due to technical issues, we were not able to track 3 participants’ app use in cohort 3. Therefore, data analysis on app engagement in cohort 3 was based on 82 (96%) of 85 participants. In general, participants perceived almost all the features to be useful. The top 5 most liked features of the app included “quit plan,” “tracking,” “reminders and notifications,” “MOtalks,” and “motivational quotes.” MOtalks are short video or audio lectures of a specific theme related to smoking cessation. Participants explained that the “quit plan” feature “helped with learning about the habit and situations that trigger me to smoke and how to make a plan to deal with it,” and the “tracking” feature “showed me some patterns I wasn’t aware of.” Participants liked the “reminders and notifications” because “the daily reminders came as pop-up notifications to enter your smoking status for the day. With that it makes you feel responsible and like you are making progress.” Participants also enjoyed the “quotes” because they were “uplifting” and “motivational.” These findings were consistent with users’ actual engagement with various features, with “login” as the most frequently used function, followed by “quit plan” and “medication tracker.” The least liked feature of the app was the “comments.” Because this feature was not frequently used, participants mentioned that “I could not see any comments or messages there.”

**Table 3:** Use of the MO app.

		Cohort 1	Cohort 2	Cohort 3
**General use of the app, mean (SD)**				
	Duration of app use (days)	37.56 (29.79)	35.33 (36.56)	46.31 (41.52)
	Total activities engaged (times)	89.22 (76.62)	159.60 (213.97)	158.89 (236.71)
	Unique activities engaged (types)	37.11 (23.85)	79.42 (76.45)	67.66 (80.83)
	Log-in times	17.56 (21.38)	13.75 (28.57)	9.98 (15.79)
	Points earned	20,430 (25916.20)	26,933.75 (33859.66)	28,764.48 (40,157.13)
**Engagement with specific app features (most frequently engaged features), mean score**				
	Quit plan	8.00	6.46	12.47
	Medication tracker	4.89	3.00	3.56
	MOtalks: meet your hosts	0.89	4.5	2.25
	Why quit	1.11	0.83	2.20
	MOtalks: denial stories	0.22	2.33	2.12
	MOtalks: triggers	0.33	1.00	1.85
	Quit date setting	1.11	2.75	1.74

### Suggestions for Improvement

The following themes emerged from thematic analysis of participants’ responses to open-ended questions regarding things to be improved for the app. The first identified theme was “availability.” Participants recommended “enabling it in all devices—that is, iOS and Android.” The second theme was “staying logged in.” Participants mentioned that “there should be an option to always stay logged in since no one else uses my phone but me. The ability to stay logged in after closing the app for a while will save me the time of having to log in with my details again or entering my phone passcode since my phone is virtually always on me.” “Stability” was the third theme, as participants suggested to “make the app more stable and working perfectly to avoid it crashing*.*” The last theme was “removing visual smoking triggers.” As participants put it, “the app is perfect as it is, but taking out pictures of cigarettes and someone smoking would help a lot.” In addition to the general themes, participants also pointed out that certain features could be improved. For example, for the tracking function, participants could not see all the days they smoked even within that month. Participants also think the “common questions” feature “needs more development and more options to pick from*.*”

### Preliminary Effectiveness

We performed a series of logistic regression analyses to examine whether participants with greater app engagement are more likely to self-report quitting at the 6 and 12-week follow-ups (hypothesis 1). Results showed a significant effect of total activities engaged on both 6-week cessation status (Wald *χ*^2^_1_=4.41; *P*=.04) and 12-week cessation status (Wald *χ*^2^_1_=7.93; *P*=.005). The odds of staying abstinent at week 6 and week 12 increased by 0.3% (odds ratio [OR] 1.003, 95% CI 1.00-1.01) and 0.4% (OR 1.004, 95% CI 1.001-1.01), respectively, for every additional app activity engaged.

The types of activities participants engaged in also significantly predicted the odds of quitting at 6 weeks (Wald *χ*^2^_1_=4.19; *P*=.04) and 12 weeks (Wald *χ*^2^_1_=7.97; *P*=.005). The odds of staying abstinent at week 6 and week 12 increased by 0.6% (OR 1.006, 95% CI 1.00-1.01) and 0.9% (OR 1.009, 95% CI 1.003-1.02), respectively, for every additional type of activity engaged.

Use frequency, indicated by “login times,” did not significantly influence smoking cessation rate at 6 weeks (Wald *χ*^2^_1_=1.85; *P*=.17, OR 1.02, 95% CI 0.99 to 1.06) but predicted the odds of quitting at 12 weeks (Wald *χ*^2^_1_=5.25; *P*=.02). The odds of maintaining abstinence from smoking at week 12 increased 1.05 times for each additional log-in (OR 1.05, 95% CI 1.01-1.08).

To examine whether earning more points is associated with a higher likelihood of self-reported quitting (hypothesis 2), we recoded participants’ points earned into 4 dummy coded categories: >5000 points, >10,000 points, >50,000 points, and >100,000 points. Among them, only “>5000 points” significantly predicted the odds of quitting at 6 weeks (Wald *χ*^2^_1_=5.96; *P*=.02) and 12 weeks (Wald *χ*^2^_1_=10.41; *P*=.001). Participants who earned >5000 points had higher odds of quitting at 6 weeks (OR 3.12, 95% CI 1.25-7.75) and 12 weeks (OR 4.65, 95% CI 1.83-11.76). Changes in other smoking-related outcomes from baseline, 6 weeks, to 12 weeks in cohort 3 were summarized in Table 4.

**Table 4:** Smoking-related outcomes in cohort 3 (N=85).

Characteristic		Baseline upon installation, n (%)	6 weeks, n (%)	12 weeks, n (%)
**Number of participants completed the survey**				
	Completed	85 (100)	56 (66)	47 (55)
	Missed	0 (0)	29 (34)	38 (45)
**Smoking status**				
	Active smoker	85 (100)	8 (9)	5 (6)
	Quit	0 (0)	42 (49)	40 (47)
	Not sure	0 (0)	6 (7)	2 (3)
**Abstinent from smoking in the past 7 days**				
	Yes	—^a^	49 (58)	44 (52)
	No	—	7 (8)	3 (4)
**Smoking in the past 30 days**				
	None of the days	—	25 (29)	31 (36)
	A few days	—	24 (28)	12 (14)
	Some days	—	5 (6)	3 (4)
	Most days	—	2 (26)	1 (1)
**Cigarettes smoked per day (on average)**				
	0 cigarette/d	0 (0)	48 (56)	42 (49)
	1 cigarette/d	14 (16)	2 (26)	1 (1)
	2-4 cigarettes/d	28 (33)	4 (5)	4 (5)
	5-10 cigarettes/d	24 (28)	2 (2)	0 (0)
	11-20 cigarettes/d	11 (13)	0 (0)	0 (0)
	21-30 cigarettes/d	8 (9)	0 (0)	0 (0)
**FTND score^b^ (0-10 scale)**				
	Very low (0-2)	7 (8)	6 (7)	3 (4)
	Low (3-4)	34 (40)	1 (1)	1 (1)
	Moderate (5)	17 (20)	1 (1)	1 (1)
	High (6-7)	18 (21)	0 (0)	0 (0)
	Very high (8-10)	8 (9)	0 (0)	0 (0)

^a^Not available.

^b^FTND: Fagerstrom Test for Nicotine Dependence. This score was only assessed and analyzed among active smokers.

We also performed a series of post hoc analyses to explore the effects of specific app features on cessation outcomes. In the prequit and planning phase, engaging with “why track,” “what influences are around me,” “quit story,” “meducation vaping,” or “meducation nicotine addiction” predicted a greater likelihood of quitting at both week 6 and week 12. Completing reasons for “why quit” and “MOtalks triggers” did not affect the 6-week cessation status but was significantly associated with participants’ 12-week cessation status. Completing “about me” was not linked with cessation status at either time point. Detailed results are reported in Table 5.

**Table 5:** Effects of engaging with specific app features on cessation outcomes.

		Wald chi-square	*P* value	OR^a^ (95% CI)
**Quit at week 6**				
	About me	0.19	.67	1.31 (0.39-4.94)
	Why quit	2.39	.12	2.05 (0.83-5.07)
	Why track	4.35	.04	2.75 (1.06-7.12)
	Quit story	4.53	.03	2.71 (1.08-6.76)
	What influences are around me	5.05	.03	3.19 (1.16-8.75)
	MOtalks triggers	3.39	.07	3.15 (0.93-10.69)
	Meducation vaping	3.94	.05	2.93 (1.01-8.47)
	Meducation nicotine addiction	5.12	.02	3.63 (1.19-11.12)
	Medication tracker	3.57	.06	2.66 (0.96-7.36)
	MOtalks medication	0.82	.37	1.72 (0.53-5.58)
	MOtalks breathing	2.61	.11	2.42 (0.83-7.07)
	MOtalks urges	0.64	.42	1.57 (0.52-4.76)
	MOtalks stress	4.09	.04	3.50 (1.04-11.79)
	MOtalks tools	0.82	.37	1.72 (0.53-5.58)
**Quit at week 12**				
	About me	3.05	.08	3.10 (0.87-11.06)
	Why quit	5.79	.02	3.22 (1.24-8.36)
	Why track	6.39	.01	3.33 (1.31-8.48)
	Quit story	7.67	.006	3.66 (1.46-9.16)
	What influences are around me	6.40	.01	3.50 (1.33-9.24)
	MOtalks triggers	6.94	.008	5.20 (1.52-17.74)
	Meducation vaping	4.41	.04	2.94 (1.07-8.02)
	Meducation nicotine addiction	5.47	.02	3.45 (1.22-9.73)
	Medication tracker	8.83	.003	4.76 (1.70-13.31)
	MOtalks medication	2.91	.09	2.79 (0.86-9.05)
	MOtalks breathing	6.64	.01	4.13 (1.40-12.15)
	MOtalks urges	2.80	.09	2.58 (0.85-7.83)
	MOtalks stress	8.01	.005	5.83 (1.72-19.78)
	MOtalks tools	2.91	.09	2.79 (0.86-9.05)

^a^OR: odds ratio.

In the quit and maintenance phase, we found a marginally significant effect of using the medication tracking feature on the 6-week cessation status (Wald *χ*^2^_1_=3.57; *P*=.06; OR 2.66, 95% CI 0.96-7.36), and a significant effect on the 12-week cessation status (Wald *χ*^2^_1_=8.83; *P*=.003; OR 4.76, 95% CI 1.70-13.31). Similarly, participants who completed “MOtalks breathing” had a greater chance of quitting at the 12 weeks than those who did not. Engaging “MOtalks medication” was not associated with cessation outcomes.

In the relapse prevention phase, learning from “MOtalks stress” was significantly linked with a higher likelihood of quitting at 6 and 12 weeks. However, engaging with “MOtalks urges” or “MOtalks tools” did not predict cessation status.

## Discussion

### Overview

In this study, we used a systematic approach to develop and pilot-test the MO mobile phone app for smoking cessation. This process involved iterative engagement with users, incorporating their feedback to make improvements and updates to the app. We used a combination of qualitative and quantitative methods, including closed and open-ended questions and app use tracking, to gather user feedback and refine the app’s features.

In cohort 1, we identified and fixed the issue of frequent crashes and enhanced the point reward system by adding badges. We also tailored the progress reports and reminders based on users’ stages of quitting. On the basis of user data analysis in cohort 2, we further refined the MO app by introducing a new “chat” feature, which served as an internal support group to replace a prior support group feature that required participants to exit the app to engage it. Discussions in this chat were moderated by a researcher on an intermittent basis. In addition, we implemented a new reward mechanism where users achieving the intermediate stage (5 stages in total: beginner, fast starter, intermediate, master, and expert) of 20,000 points were entered into a lottery for a US $50 gift card. These updated features aimed to promote engagement and motivation among participants to quit smoking. The performance of the app steadily increased from cohort 1 to 3, with cohort 3 reporting the highest level of acceptability and engagement, as indicated by the duration of active app use and total points earned. The “quit plan” feature was the most frequently used among cohort 3 participants. Participants also frequently interacted with and positively rated the MO talks. The most popular topics of MO talks were “meet your host,” “denial stories,” and “breathing.”

### Principal Findings

Results demonstrated that the MO app was promising in assisting smokers to quit. The 7-day point prevalence abstinence rates of the mITT population were 58% (49/85) at week 6 and 52% (44/85) at week 12, while the 30-day point prevalence abstinence rates were 29% (25/85) at week 6 and 36% (31/85) at week 12. These cessation rates were higher than the 7-day point prevalence abstinence rates reported for other smoking cessation apps, which typically ranged from 13% to 35% at the 12-week follow-up [9,16,24]. We also found that participants who engaged more frequently with the app’s features, interacted with a greater variety of features, and earned more points were more likely to maintain abstinence from smoking. Participants that did not complete the study questionnaire were imputed to return to smoking. This conservative approach may not capture any benefits, such as decreased cigarette use for a period. Furthermore, no adverse events or safety and privacy concerns were reported during the study period. Despite minor technical issues, such as unexpected crashes, all features of the app functioned as intended, indicating its feasibility for everyday use by smokers.

The significant association between app engagement and cessation outcome found in this pilot study underscores the critical role of an engaging system in driving and sustaining abstinence. Previous research has also shown that improved abstinence rates were linked with increased app engagement, indicated by a greater number of app openings and adherence to app features (ie, completing required tasks) [16,27,28]. In a pilot randomized clinical trial, the number of interactions with app features completely mediated cessation outcomes [29]. Our study contributes to existing literature by identifying the types of activities users engaged in and the points earned through interactions with app features as additional forms of engagement that significantly predict smoking cessation outcomes. We found that users who received >5000 points were more likely to remain abstinence at weeks 6 and 12. However, users who accumulated >10,000 points did not show significantly different cessation outcomes compared with those with fewer points. This suggests that earning 5000 points represents a critical threshold for the app’s effectiveness. The finding that additional points beyond this threshold did not enhance cessation outcomes does not necessarily indicate a ceiling effect but rather reflects the complex and recursive nature of smoking cessation. Users who found quitting relatively easy may not need to earn many points and can quit at an early stage. In contrast, users who accumulated >10,000 points might indicate 2 possibilities: on one hand, they were highly motivated to quit and thus engaged more with the app; and by contrast, they faced greater challenges in quitting, requiring prolonged app engagement. While both scenarios result in greater points earned, the former is associated with more favorable quitting outcomes, whereas the latter is linked to continued smoking or an extended period required to quit.

Our data shed light on several strategies for designing engaging health apps. First, smoking cessation apps should provide users with the flexibility to create their own quitting plans, including setting quit dates, identifying reasons to quit, establishing quitting goals, and learning about medication options. These features were the most frequently used in the MO app. Second, the app should enhance users’ efficacy in quitting smoking and equip them with skills to cope with relapses. Features that facilitated the tracking of smoking habits, sent reminders, and provided educational materials on strategies for managing urges were rated as the most liked features by participants. These design considerations align with the self-determination theory [30,31], which suggests that behaviors are driven by the internal needs of autonomy (ie, taking control of the quitting process), competence (ie, having necessary skills to achieve the cessation goal), and relatedness (ie, social connections and sense of belonging). Features related to “autonomy” and “competence” were well received by MO users. While MO also addressed the “relatedness” need by establishing a secure peer-support community within the app through chat and story sharing functions, these features were not completely used by participants. This finding is consistent with previous research that showed low levels of engagement and perceived usefulness for smoking cessation apps that relied on social network members [32]. One potential reason for such limited engagement is the challenge participants faced in building trust with other users within a relatively short period. However, the chat and story booth features may play a more important role for users in the later stage of smoking cessation, especially when they experience relapses. In addition to sharing experiences with smoking cessation peers, individuals may also need support and encouragement from family members or close friends. While receiving social support has been positively associated with changes in smoking cessation stages [33], meta-analyses yielded mixed results regarding the effects of interventions that enhance partner and family support on cessation rates [34,35]. Therefore, stronger empirical evidence is needed to determine the effectiveness of integrating family-based support into the design of smoking cessation apps.

In response to open-ended questions soliciting suggestions for increasing app engagement, participants emphasized the importance of reducing barriers to app use and making it as accessible as possible. Recommendations included streamlining the log-in process and ensuring compatibility with both iPhone and Android devices. Enabling logins with biometrics, such as using fingerprints or facial recognition, would further enhance the convenience and security of the log-in process. Participants were also inspired by the motivational talks and uplifting quotes provided by the app, as these elements helped build confidence and foster positive beliefs about quitting smoking. On the basis of this finding, it might be helpful to further refine the “story booth” function, including providing prompts, instructions, or incentives to encourage former successful quitters who used the app to record their experience, allowing different forms of story sharing (eg, textual, audio, or video stories), and helping current users find quitters sharing similar backgrounds with them so that they are more likely to learn from and become motivated by peers’ quitting journey. Furthermore, the point system and financial incentives were found to be useful in increasing participants’ motivation to remain engaged with the MO app. The financial incentives may be particularly attractive to and useful in assisting low-SES smokers in quitting. These reward mechanisms could be reinforced in future iterations and adapted to nonstudy users. For example, the point system could be used to unlock advanced features (eg, personalized motivational quotes, an artificial intelligence-powered smoking cessation chatbot). In addition, enabling users to share their achievements on social media or with significant others could further enhance engagement and motivation. While it may not be feasible to provide large-scale monetary rewards to public users, alternative financial incentives could be offered in the form of free web-based consultation with professionals, free nicotine cessation medications, or coupons for healthy products.

Given the proliferation of numerous cessation apps with varying quality, clinical research data are essential to help smokers make informed choices and enhance their chances of success. Although there has been an increase in clinical research on mobile phone-based smoking cessation interventions in recent years, we are still in the early stages of accumulating rigorous clinical research evidence to establish best practices for smoking cessation apps. Health care professionals require credible data to confidently recommend mHealth platforms to their patients. Our findings were based on user experience and cessation outcomes of a good mix of males and females as well as smokers of different ethnicities and races, representing a diverse smoking population. By identifying users’ level of app engagement (eg, 5000 points) associated with cessation, the app platform can serve as a tool for health care professionals to encourage patients to reach and surpass this activity level. Moreover, this mobile-based system can be easily integrated into clinical practices, such as short advice or counseling sessions, complementing clinical support for smoking cessation and assisting in tracking patients’ adherence to cessation treatment.

### Limitations

The study had several limitations. First, being a pilot evaluation study, the sample size was small, and the representativeness of the sample cannot be guaranteed. Therefore, the findings may not be generalizable to the entire population of smokers intending to quit. Although recruitment through web-based advertisements proved to be feasible and efficient in recruiting eligible participants, it is important to note that the participants in our study exhibited high levels of technology literacy and motivation to quit smoking. Participants who joined this research were young, with more than 85.8% (91/106) of participants aged younger than 40 years, and most of them had a smoking history between 1 and 10 years. We also set an eligibility criterion requiring participants to have a high intention to quit (ie, scored at least a 7 on a scale of 0-10 on a self-assessed commitment scale). The unique characteristics of the sample, including the younger age, relatively short smoking history, and strong motivation to quit, could be reasons for the high smoking cessation rate of using the MO app. In addition, the study was conducted during the COVID-19 pandemic, which presented a unique opportunity for smokers to quit due to social distancing and quarantine policies that significantly reduced social and environmental smoking triggers. This health crisis also made staying healthy a more salient goal in people’s minds, and therefore, participants may have been more motivated to quit smoking. By contrast, social isolation, anxiety, and depression increased during the pandemic, and self-reported cessation attempts and cessation-related medications declined at the beginning of the pandemic [36]. The unique characteristics of participants, social contexts, and the special period should be considered when interpreting this study’s findings.

Second, the follow-up period in this study was relatively short (6 and 12 weeks) for testing the app’s engagement and effectiveness. Smoking cessation is a long-term process involving potential setbacks and relapses. In future studies, we plan to follow-up with app users for 6 months and 1 year to examine the apps’ long-term cessation effects and the dynamic relationship between app engagement and cessation outcomes at different stages of quitting.

Third, this study assessed cessation outcomes through self-reported data from web-based surveys, without biochemical verification of cotinine levels or exhaled carbon monoxide monitoring. Obtaining biochemically verified cessation status was not feasible during the pandemic period, and the Society for Research on Nicotine and Tobacco Subcommittee on Biochemical Verification has suggested that biochemical confirmation may not be necessary in certain studies [37]. Considering cost and other constraints, self-reporting can be considered an acceptable standard method in cessation trials and clinical practice [38]. However, future studies incorporating biochemically verified data would be valuable to check whether the high cessation rate after using the MO app can be replicated.

Finally, this study did not have time-stamped historical logs of user engagement with each app feature, so we were unable to demonstrate users’ dynamic engagement with specific activities over time. To improve the user-centered design and usability of the app, it is critical to understand how users interact with the app initially, whether they can quickly learn and explore the various functions, and how users’ initial, instant responses to the app differ from adaptive responses several weeks later. It is useful for future studies to track users’ first-week use and compare it with use at 1 month.

One advantage of the MO app is that it incorporates the medication tracking function to facilitate smokers with medication use and compliance of cessation medications in everyday settings. Due to the small sample size, we were only able to analyze the effect of using the medication tracking function on cessation outcomes and were underpowered to have more detailed descriptions about how they used this function, what kinds of medications they used, and the corresponding effects. Further research should fill this gap by providing empirical evidence on how using different cessation medications and adherence rates, facilitated by smoking cessation apps, are associated with cessation outcomes.

Our next step is to conduct a larger-scale randomized clinical trial that compares MO with other standard smoking cessation apps to further explore its effectiveness on short-term and long-term quitting outcomes. It is also worth investigating whether the app is accessible to and acceptable to low-SES smokers, which is critical to help reduce disparities in smoking cessation. While mHealth apps have enhanced the accessibility of health interventions to the public, it is unclear whether disparities exist in the use of mobile technologies for smoking cessation. Considering that the low-SES population comprises most current smokers, understanding the effects of MO on facilitating smoking cessation among this group is necessary.

### Conclusions

MO is a feasible mobile phone app that effectively provides smoking cessation support to individuals who desire to quit. The study demonstrated high acceptability, usability, and potential efficacy of the app in enhancing cessation outcomes. Further research, including a randomized controlled trial, is needed to rigorously evaluate long-term app engagement and effects on smoking cessation.

## Appendix

Multimedia Appendix 1 A walkthrough video explaining the main features of the MO smoking cessation app.

## Abbreviations

ITT: intent-to-treat
mHealth: mobile health
mITT: modified intent-to-treat
OR: odds ratio
RQ: research question
SES: socioeconomic status
